# Novel stirring method for small-scale dissolution test: Rotating vessel method

**DOI:** 10.5599/admet.3136

**Published:** 2026-01-11

**Authors:** Shiori Ishida, Samuel Lee, Balint Sinko, Karl Box, Kiyohiko Sugano

**Affiliations:** 1 Molecular Pharmaceutics Lab., College of Pharmaceutical Sciences, Ritsumeikan University, 1-1-1, Noji-higashi, Kusatsu, Shiga 525-8577, Japan; 2 Pion Inc. (UK) Ltd. Forest Row Business Park, Station Road, East Sussex, RH18 5DW, United Kingdom

**Keywords:** Hydrodynamics, precipitation, magnetic stirrer, ibuprofen, carbamazepine

## Abstract

**Background and purpose:**

In the compendial dissolution test, the overhead rotating paddle method (ORP) has been used for stirring, whereas the magnetic stirring bar method (MSB) has been employed for small-scale dissolution tests, such as the μDISS Profiler^TM^ (μDISS). Previous reports have indicated that differences exist in the precipitation profiles of a drug between ORP and MSB, because the latter causes contact-induced nucleation. However, it has been difficult to use an ORP and an *in situ* UV probe simultaneously in μDISS. In this study, a novel stirring method, the rotating vessel method (RV), was developed for μDISS. The dissolution and precipitation profiles of model drugs in RV-μDISS were then compared with those in MSB-μDISS, as well as with the results of conventional dissolution tests using an ORP.

**Experimental approach:**

In RV-μDISS, a small paddle (approximately 1/4 of the conventional paddle) was fixed to the UV probe, and the glass vessel was rotated to produce a flow pattern similar to that of ORP. The dissolution and bulk-phase precipitation tests were performed for ibuprofen sodium (IBU Na) and carbamazepine (CBZ), respectively, using RV-μDISS and MSB-μDISS, as well as ORP with the conventional vessel (500 mL, for IBU Na) (CV) or the mini-vessel (50 mL, for CBZ) (MV).

**Key results:**

The dissolution rate of IBU Na was similar in all methods. Rapid precipitation of crystalline IBU free acid was observed in the MSB-μDISS method. In contrast, no crystalline precipitation was observed in RV-μDISS and ORP-CV, and the drug phase-separated as a liquid (oil) phase (liquid-liquid phase separation). The precipitation rate of CBZ in RV-μDISS was similar to that in ORP-MV, but slower than that in MSB-μDISS.

**Conclusion:**

The precipitation profile in RV-μDISS was close to those in ORP-CV and ORP-MV. RV-μDISS would be a useful tool for the assessment of the precipitation profiles of drugs.

## Introduction

Recent drug candidates tend to be poorly soluble in aqueous media [1,2]. Poor aqueous solubility can cause poor and variable oral bioavailability of a drug. To overcome the poor solubility, drug candidates are often formulated as supersaturable drug substances and formulations such as salts, cocrystals, and amorphous solid dispersions [3-5]. Therefore, it is important to evaluate the dissolution and precipitation profiles of drug candidates appropriately during drug discovery and development. Since the amount of a drug candidate available is limited (typically <<1 g), small-scale dissolution tests such as μDISS Profiler^TM^ (μDISS) are widely used in drug discovery and early drug development. In a small-scale dissolution test, a magnetic stirring bar method (MSB) is mostly used for stirring rather than an overhead rotating paddle method (ORP).

It is well known that stirring methods can have a dramatic impact on the dissolution and precipitation profile of a drug [6]. When compared to ORP, MSB would induce secondary nucleation more pronouncedly by friction between the stirring bar and the vessel [7]. Since there is no such friction in the gastrointestinal tract, the use of MSB can potentially overestimate precipitation in vivo [6]. In MSB-μDISS, the agitator geometry has a significant effect on the precipitation profiles of danazol due to differences in the hydrodynamic shear produced by the agitation [8]. Recently, Zöller *et al.* [9] reported that the precipitation of ibuprofen in MSB-μDISS was significantly faster than in the compendial ORP dissolution test. In MSB-μDISS, the friction between the magnetic stirring bar and the vessel bottom was suggested to enhance the nucleation process of crystalline ibuprofen free acid [9]. However, it has been difficult to insert an overhead paddle and an *in situ* UV probe simultaneously into a small vessel.

**Figure 1.**
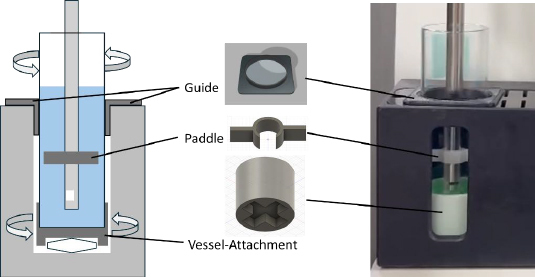
Rotating vessel method.

The purpose of the present study was to develop a novel stirring method for a small-scale dissolution test. In this study, the rotating vessel method (RV) was newly developed for μDISS (RV-μDISS) (Figure 1). The dissolution and bulk-phase precipitation tests were performed for ibuprofen sodium (IBU Na) and carbamazepine (CBZ), respectively, using RV-μDISS and MSB-μDISS, and compared with the conventional ORP methods.

## Experimental

### Material

Ibuprofen free acid (IBU FA), ibuprofen sodium (IBU-Na), carbamazepine anhydrous form (CBZ AH), sodium dihydrogen phosphate dihydrate, sodium chloride, 6 N hydrochloric acid, acetonitrile, and dimethylacetamide (DMA) were purchased from FUJIFILM Wako Pure Chemical Corporation (Osaka, Japan).

### Methods

#### Development of the rotating vessel method

The μDISS Profiler^TM^ (Pion Inc., Billerica, USA) was used in this study. Figure 1 shows the RV-μDISS method. A small paddle was created by cutting a polyoxymethylene plate using a computer numerical control slicer (20 mm, approximately 1/4 of the conventional paddle) (ORIGINALMIND Inc., Nagano, Japan). The attachment and guides for the vessel were created from ABS resin using a 3D printer (ADVENTURE 5M Pro, APPLE TREE Co., Ltd, Osaka, Japan). The paddle was fixed to the UV probe. The magnetic stir bar (20 mm long) was attached beneath the vessel bottom. The vessel guide was mounted on the top of the μDiss device to prevent the vessel from wobbling.

#### Dissolution test using the μDiss™ apparatus

Dissolution tests using RV-μDISS and MSB-μDISS were performed at 37±0.5 °C. The Japanese Pharmacopoeia first fluid (pH 1.2 HCl, 34 mM NaCl) (JP1) and a pH 1.2 HCl solution without NaCl (pH 1.2 HCl) were used as test media. Each test medium (15 mL) was added to a vessel. A cross-prim magnetic stirring bar was used for MSB-μDISS (20 mm size). The rotating speed was set at 75 or 150 rpm for both RV-μDISS and MSB-μDISS. IBU-Na (6.64 mg) was added to each vessel. The drug concentration was continuously monitored using the UV probe with 2 mm apertures at 260 nm. Dissolution tests were performed in triplicate. The residual solids were collected by vacuum filtration, and the solid form was determined as described below.

#### Precipitation test using the μDiss™ apparatus

Bulk phase solvent-shift precipitation tests were performed for carbamazepine (CBZ) using RV-μDISS and MSB-μDISS at 37 ± 0.5 °C. The fasted state simulated intestinal fluid without bile micelles (blank FaSSIF) was used as a test medium (29 mM phosphate, 105 mM NaCl, pH 6.5). The rotating speed was set at 100 rpm for both RV-μDISS and MSB-μDISS. Carbamazepine anhydrous form (CBZ AH) was dissolved in DMA (240 mM, 0.15 mL) and then added to the blank FaSSIF (15 mL). The drug concentration was continuously monitored using the UV probe with a 2 mm aperture at 320 nm. Precipitation tests were performed in triplicate. The precipitates were collected by vacuum filtration, and the solid form was determined as described below.

#### Dissolution test using the USP apparatus 2

The dissolution tests of IBU Na were performed with the USP apparatus 2 at 37±0.5 °C (ORP with a conventional vessel (ORP-CV)). JP1 and pH1.2 HCl were used as test solutions. Each test solution (500 mL) was prewarmed, degassed, and added to a vessel. The paddle rotating speed was set to 75 rpm. IBU-Na (221.3 mg) was added to each vessel. A sample solution (1 mL) was withdrawn at specified time intervals and immediately filtered through a hydrophilic PVDF filter (*ϕ* = 4 mm, pore size: 0.22 μm, Merck). The first few droplets were discarded to avoid filter adsorption. The filtrate (750 μL) was diluted with acetonitrile (250 μL). The drug concentration was determined by HPLC (Shimadzu Prominence LC-20 series and Agilent Technologies 1200 Series, column: ZORBAX Eclipse Plus (C18 2.1×50 mm, 3.5 μm) (Agilent Technologies), flow rate: 0.6 mL min^-1^, mobile phase: 0.1 % trifluoroacetic acid-acetonitrile/0.1 % trifluoroacetic acid-water 50 %, detection: UV 260 nm, column temperature: 40 °C, and injection volume: 10 μL). After the dissolution test, the precipitates were collected by vacuum filtration, and the solid form was determined as described below. All tests were performed in triplicate.

#### Solid form determination

The solid forms of residual particles and precipitants were determined by powder X-ray diffraction (PXRD) and differential scanning calorimetry (DSC). Before PXRD analysis, the collected samples were gently pulverized with a mortar and pestle. A zero-diffraction plate was used as a sample holder. PXRD data were measured from 5 to 35° (2*θ*) (scanning speed: 10° min^-1^; step size: 0.02°; Cu Kα radiation (15 mA, 40 kV)) (MiniFlex, Rigaku Corporation, Tokyo, Japan). In the DSC measurement, samples were placed in a non-sealed aluminium pan. DSC was measured under nitrogen gas at 10 °C min^-1^ (DSC60 plus, Shimadzu Corporation, Kyoto, Japan).

## Results and discussion

### Dissolution profile of ibuprofen sodium

**Figure 2.**
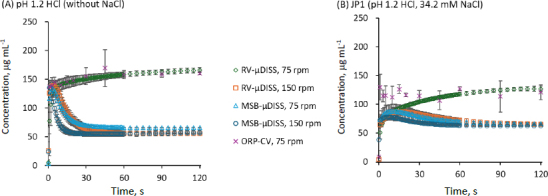
Dissolution profiles of IBU-Na in (A) pH 1.2 HCl (without NaCl) and (B) JP1 (pH 1.2 HCl containing 34.2 mM NaCl) under various stirring conditions. Mean ± S.D., *N* = 3. An enlarged view of the initial dissolution phase (0 to 30 minutes) is shown in Supplementary material Figure S1.

The dissolution profile of IBU-Na was investigated in a pH 1.2 HCl solution without NaCl (pH 1.2 HCl) and JP1 (pH 1.2 HCl containing 34 mM NaCl) under the three stirring conditions: RV-μDISS, MSB-μDISS, and ORP-CV (USP 2) (Figure 2).

All samples recovered by filtration after 120 min were identified as crystalline IBU free acid (IBU FA) by PXRD and DSC analysis, confirming the conversion from the sodium salt to the free acid (Supplementary material, Figure S2). During the dissolution tests using ORP-CV and RV-μDISS at 75 rpm, no crystalline precipitant was observed by visual inspection. IBU FA was observed to separate as an oil droplet on the surface of the aqueous medium (Supplementary material, Figure S3). Therefore, in these cases, the filtration process may have induced the crystallization of IBU FA.

In pH 1.2 HCl without NaCl (Figure 2A), the dissolution rate of IBU Na was similar in all methods (Figure 2A). The dissolved drug concentration (*C*_dissolv_) rapidly reached the maximum concentration (*C*_max_) of approximately 0.15 mg mL^-1^ (< 5 min). This *C*_max_ corresponded to the concentration at which liquid-liquid phase separation (LLPS) occurs [10]. In ORP-CV at 75 rpm, no *C*_dissolv_ decrease was observed for 2 h. In contrast, in MSB-μDISS, the rapid precipitation of crystalline IBU FA was observed after reaching *C_max_*. The final *C*_dissolv_ corresponded to the equilibrium solubility of crystalline IBU FA [10]. These results were in good agreement with the previous study [9]. In RV-μDISS at 75 rpm, no *C*_dissolv_ decrease was observed for 2 h, similar to the case of ORP-CV. When the rotation speed was increased to 150 rpm, the precipitation of crystalline IBU FA was also observed in RV-μDISS. However, the precipitation rate was lower compared to that in MSB-μDISS.

In JP1 (pH 1.2 HCl containing 34 mM NaCl) (Figure 2B), the dissolution process was slower compared to that in pH 1.2 HCl without NaCl (Figure 2B). This slower dissolution could be due to the common ionic effect of Na^+^. Like the cases in pH 1.2 HCl, at 75 rpm, the precipitation of crystalline IBU FA was observed only in MSB-μDISS. The precipitation of crystalline IBU FA occurred gradually over 30 to 40 minutes. At 150 rpm, the precipitation of crystalline IBU FA was also observed in RV-μDISS. However, *C*_max_ in RV-μDISS was higher than that in MSB-μDISS.

In RV-μDISS and ORP-CV at 75 rpm, IBU FA precipitated as an oil droplet via LLPS. LLPS can occur either on the surface of IBU Na or in the bulk phase [11,12]. This point is important for the formulation design and the prediction of *in vivo* oral drug absorption. Further investigation is required to clarify the LLPS location.

### Precipitation profiles of carbamazepine

**Figure 3.**
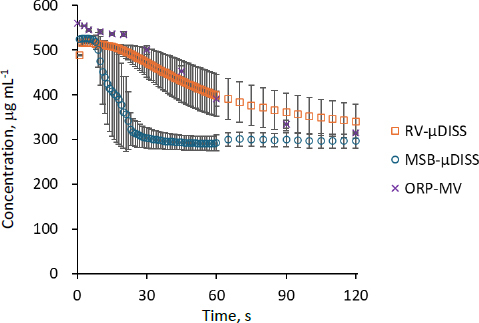
Precipitation profiles of CBZ in blank FaSSIF under various stirring conditions. Mean ± S.D., N = 3.

In this study, the bulk phase precipitation profile of CBZ was investigated using a solvent-shift precipitation test in blank FaSSIF using RV-μDISS and MSB-μDISS at 100 rpm (Figure 3). The data for ORP with a mini-vessel (50 mL scale, 100 rpm) (ORP-MV) were extracted from the previous publication [13].

All precipitates were identified as CBZ dihydrate (CBZ DH) crystals by PXRD and DSC analysis (Supplementary material, Figure S4). The drug precipitation profile in RV-μDISS was similar to that in ORP-MV, but slower than that in MSB-μDISS.

These results suggest that RV-μDISS can produce dissolution and precipitation profiles that are similar to those produced by ORP. In the RV-μDISS configuration, the fluid was agitated by rotating the vessel against the paddle fixed to the UV probe. On the other hand, in the ORP methods, the fluid was agitated by rotating the paddle against the fixed vessel. Theoretically, the relative movements of the paddle and the vessel determine the hydrodynamics of the fluid. Therefore, even though the rotating parts were different between the two methods, they can produce a similar flow pattern, resulting in similar dissolution and precipitation profiles of drugs. However, the shapes of the vessel (flat bottom vs. round bottom) and the paddle (rectangular *vs.* round-edged) were different between the RV-μDISS and the compendial dissolution test apparatus. In addition, the dissolution medium used in μDISS was not degassed, potentially inducing precipitation by air bubbles [14]. These points may have caused the difference in the variability of drug concentration between ORP and RV-μDISS. The nucleation process is probabilistic and sensitive to experimental conditions. Therefore, precipitation profiles are prone to a large variability. These points should be further investigated in the future.

## Conclusions

In conclusion, the rotating vessel method was newly developed for μDISS. RV-μDISS can avoid the friction between the magnetic stirring bar and the vessel bottom that enhances the nucleation process of crystalline drugs. RV-μDISS produced the dissolution and precipitation profiles of the model drugs similar to those in the ORP methods. RV-μDISS would be a valuable tool to evaluate the dissolution and precipitation profiles of a drug in drug discovery and early drug development.

## Supplementary material

Additional data are available at https://pub.iapchem.org/ojs/index.php/admet/article/view/3136, or from the corresponding author on request.


