# Supplementary material for: Novel stirring method for small-scale dissolution test: Rotating vessel method
Source: ADMET DMPK. 2026 Jan 11;14:3136. doi: 10.5599/admet.3136 (PMC12994599; doi:10.5599/admet.3136)
Supplement: Supplementary file 1 [file ADMET-14-3136-S1.pdf]

ADMET & DMPK **14** (2026) S3136

Open Access : ISSN : 1848-7718

http://www.pub.iapchem.org/ojs/index.php/admet

Supplementary material to

Novel stirring method for small-scale dissolution test: Rotating vessel method

Shiori Ishida[1], Samuel Lee[2], Balint Sinko[2], Karl Box[2] and Kiyohiko Sugano[1,]

[1]*Molecular Pharmaceutics Lab., College of Pharmaceutical Sciences, Ritsumeikan University, 1-1-1, Noji-higashi, Kusatsu, Shiga 525-8577, Japan*

[2]*Pion Inc. (UK) Ltd. Forest Row Business Park, Station Road, East Sussex, RH18 5DW, United Kingdom*

ADMET & DMPK **14** (2026) 3136; https://doi.org/10.5599/admet.3136

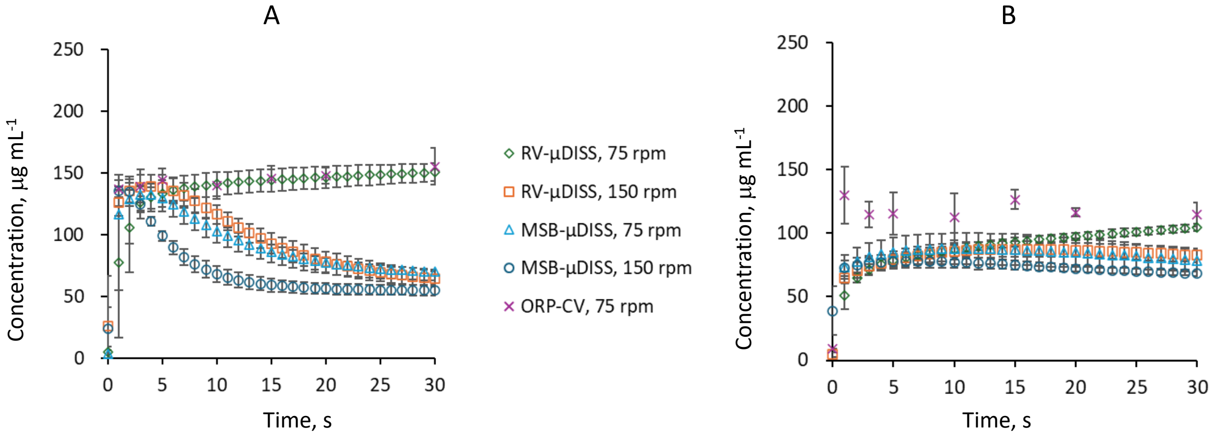

Figure S1. Dissolution profiles of IBU-Na in (A) pH 1.2 HCl (without NaCl) and (B) JP1 (pH 1.2 HCl containing 34.2 mM NaCl) under various stirring conditions (0 to 30 min). Mean ± S.D., *N* = 3.

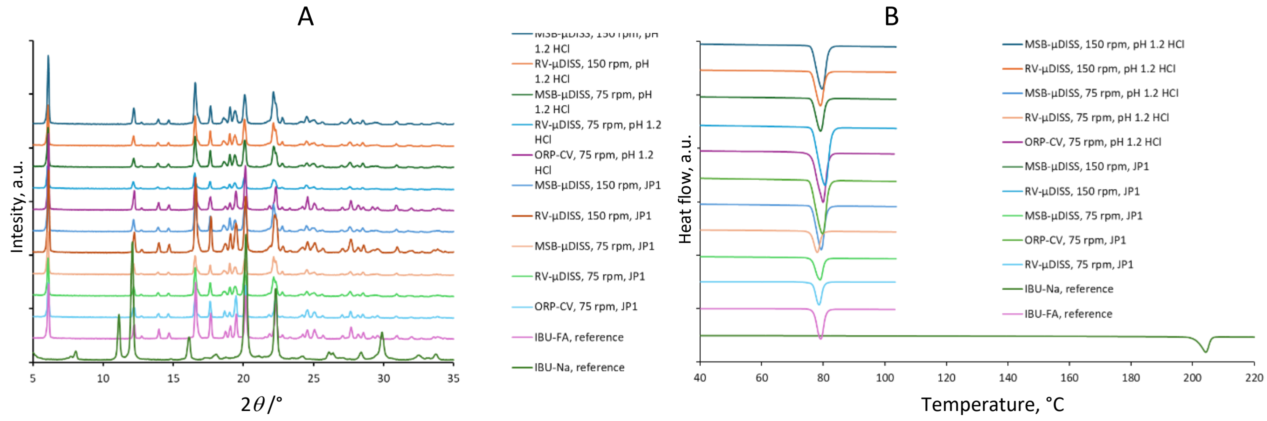

Figure S2. PXRD (A) and DSC (B) data of ibuprofen precipitants

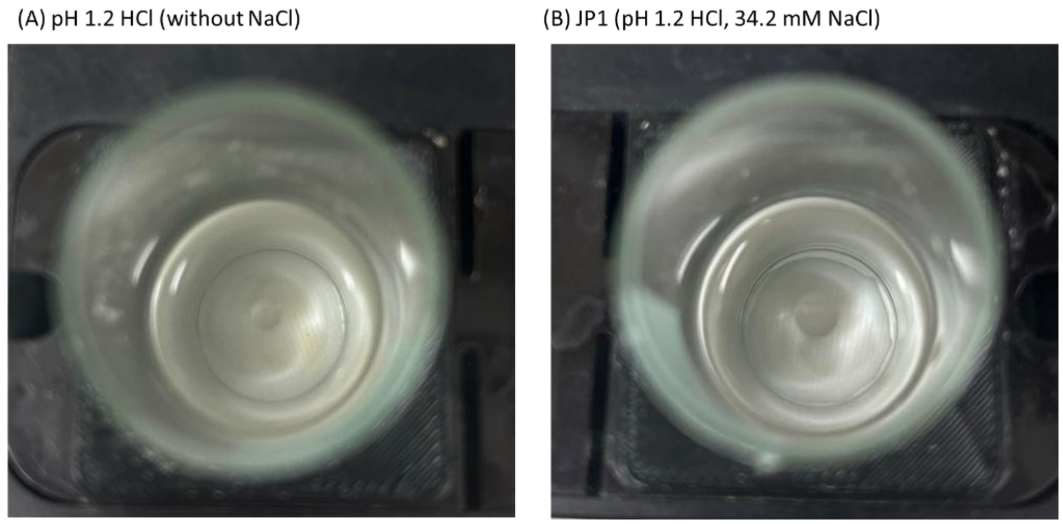

Figure S3. Oil phase separation of ibuprofen free acid

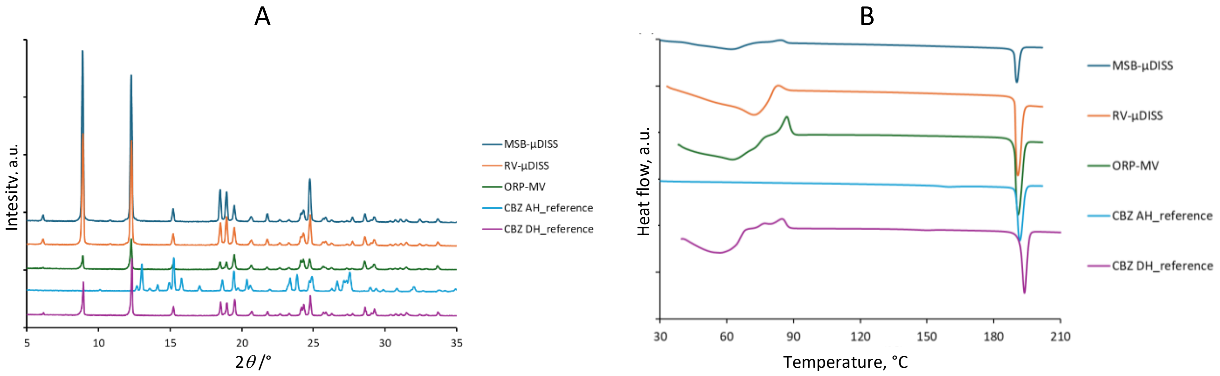

Figure S4. PXRD (A) and DSC (B) data of carbamazepine precipitants

CC BY
