# Supplementary material for: Novel stirring method for small-scale dissolution test: Rotating vessel method
Source: ADMET DMPK. 2026 Jan 11;14:3136. doi: 10.5599/admet.3136 (PMC12994599; doi:10.5599/admet.3136)
Supplement: Supplementary file 2 [file ADMET-14-3136-S1.docx]

ADMET & DMPK **14** (2026) S3136

*
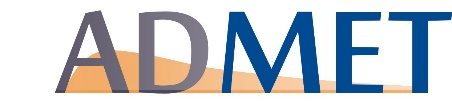
***Open Access : ISSN : 1848-7718**[***http://www.pub.iapchem.org/ojs/index.php/admet***](http://www.pub.iapchem.org/ojs/index.php/admet)

Supplementary material to

**Novel stirring method for small-scale dissolution test:
Rotating vessel method**

Shiori Ishida^1^ [
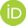
](https://orcid.org/0009-0007-4760-5006), Samuel Lee^2^ [
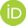
](https://orcid.org/0009-0005-4379-2807), Balint Sinko^2^ [
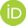
](https://orcid.org/0009-0005-8256-4348), Karl Box^2^ and Kiyohiko Sugano^1,^ [
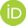
](https://orcid.org/0000-0001-5652-1786)

*^1^Molecular Pharmaceutics Lab., College of Pharmaceutical Sciences, Ritsumeikan University, 1-1-1, Noji-higashi, Kusatsu, Shiga 525-8577, Japan
^2^Pion Inc. (UK) Ltd. Forest Row Business Park, Station Road, East Sussex, RH18 5DW, United Kingdom*

ADMET & DMPK **14** (2026) 3136; <https://doi.org/10.5599/admet.3136>

A B


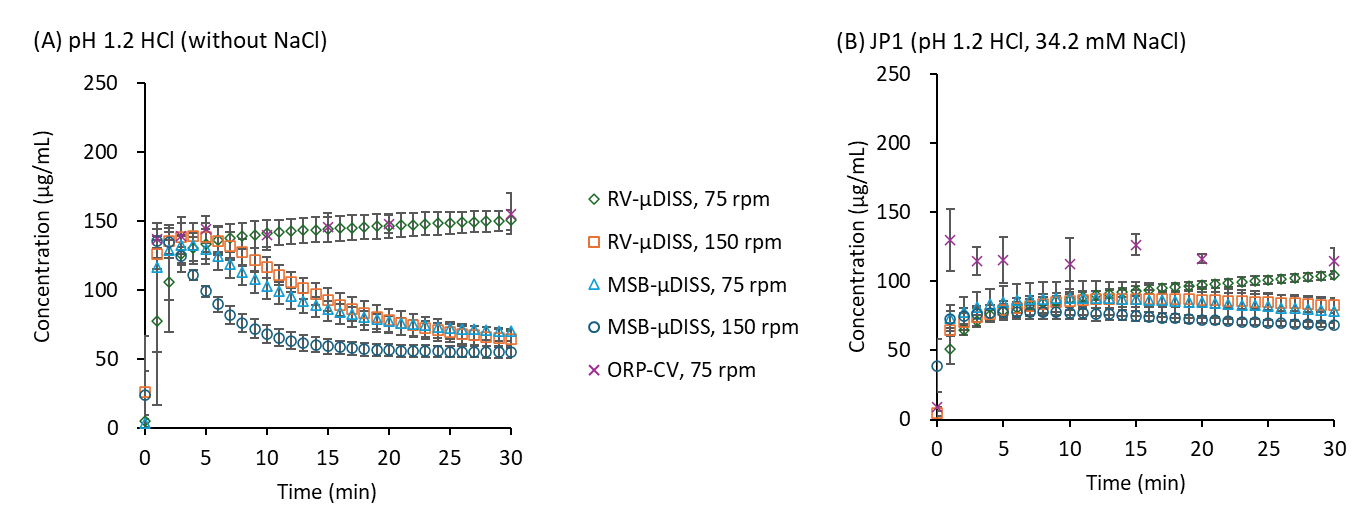

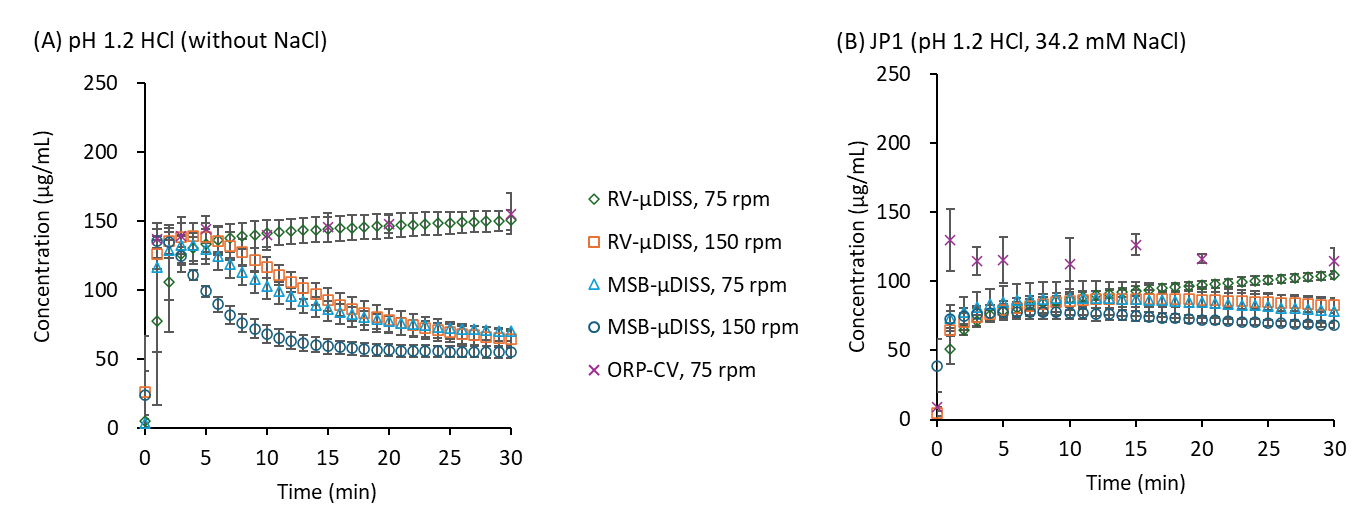


Concentration, μg mL^-1^

Concentration, μg mL^-1^

Time, s Time, s

**Figure S1.** Dissolution profiles of IBU-Na in (A) pH 1.2 HCl (without NaCl) and (B) JP1 (pH 1.2 HCl containing 34.2 mM NaCl) under various stirring conditions (0 to 30 min). Mean ± S.D., *N* = 3.

A B


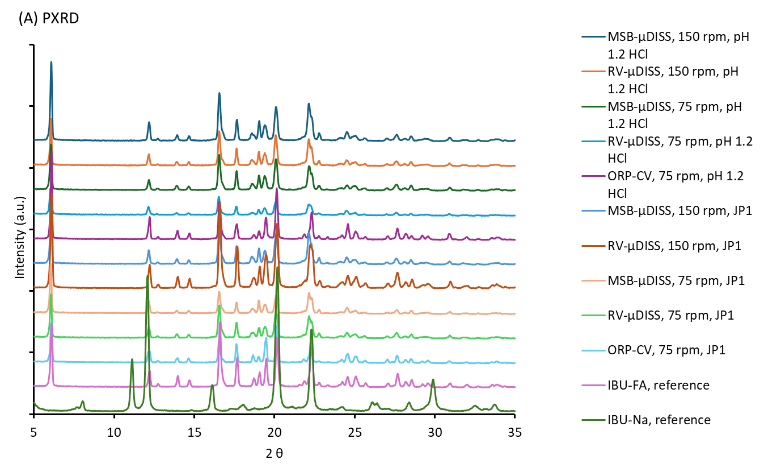

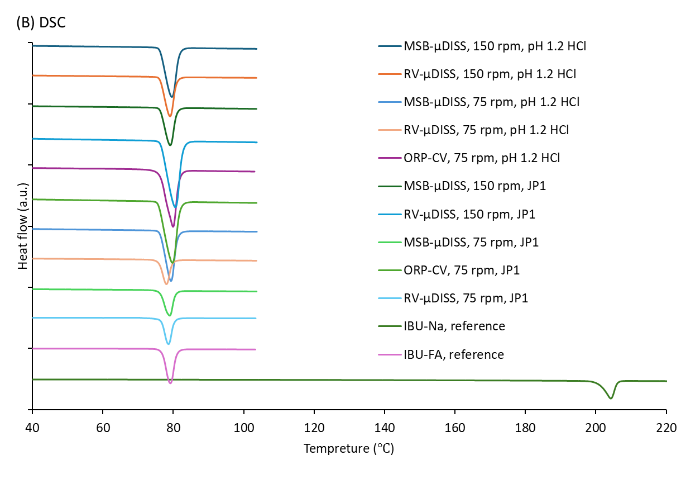


Heat flow, a.u.

Intesity, a.u.

2*θ* /° Temperature, °C

**Figure S2.** PXRD (A) and DSC (B) data of ibuprofen precipitants


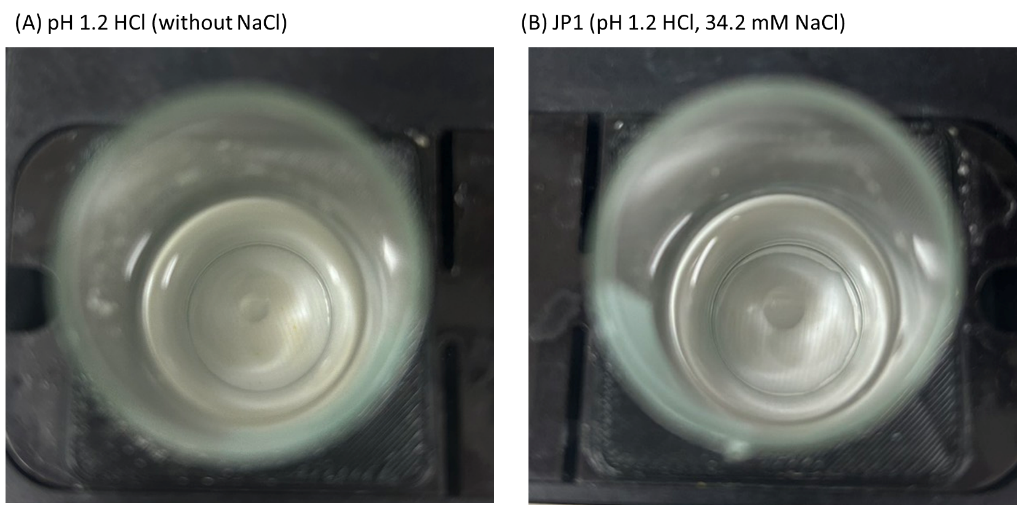


**Figure S3.** Oil phase separation of ibuprofen free acid

A B


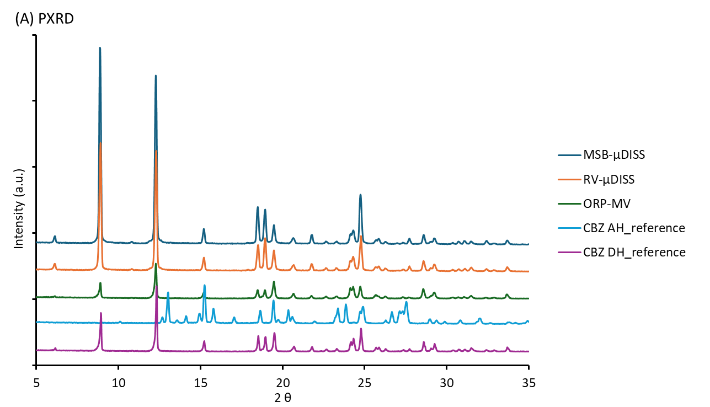

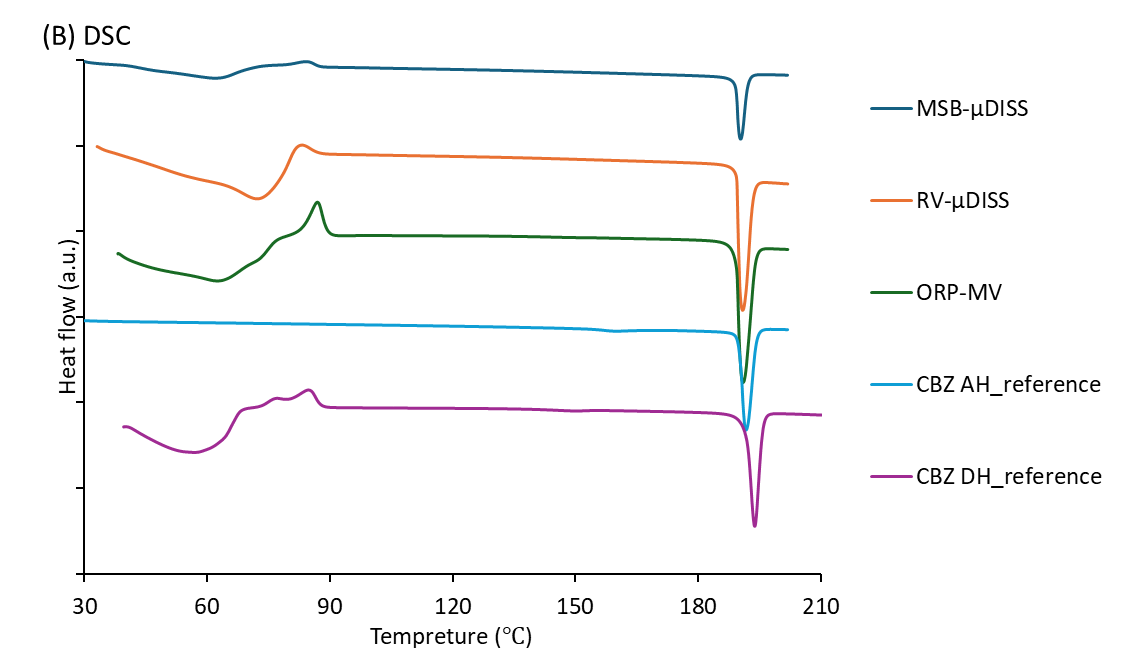


Heat flow, a.u.

Intesity, a.u.

2*θ* /° Temperature, °C

**Figure S4.** PXRD (A) and DSC (B) data of carbamazepine precipitants
